# Editorial: Innovations in MR hardware from ultra-low to ultra-high field

**DOI:** 10.3389/fphy.2022.1015289

**Published:** 2022-09-09

**Authors:** Roberta Frass-Kriegl, Lionel Marc Broche, Jean-Christophe Ginefri, Mark E. Ladd, Sigrun Roat, Mathieu Sarracanie, Simone Angela S. Winkler, Elmar Laistler

**Affiliations:** 1 Center for Medical Physics and Biomedical Engineering, Medical University of Vienna, Vienna, Austria; 2 Aberdeen Biomedical Imaging Centre, University of Aberdeen, Aberdeen, United Kingdom; 3 Université Paris-Saclay, CEA, CNRS, Inserm, BioMaps, Service Hospitalier Frédéric Joliot, Orsay, France; 4 Medical Physics in Radiology, German Cancer Research Center (DKFZ), Heidelberg, Germany; 5 Center for Adaptable MRI Technology, Department of Biomedical Engineering, University of Basel, Allschwil, Switzerland; 6 Weill Cornell Medicine, New York, NY, United States

**Keywords:** magnetic resonance imaging (MRI), ultra-low field (ULF) MRI, ultra-high field MR, MR hardware, radio frequency coils, magnet technology

Magnetic Resonance Imaging and Spectroscopy (MRI, MRS) have become indispensable diagnostic tools in numerous medical applications, providing anatomical, functional, and chemical information non-invasively with ever increasing sensitivity and specificity. However, many performance challenges are present in connection with improving the sensitivity, safety, and accessibility of these modalities. In this context, a traditional strategy to improve sensitivity is to develop ultra-high field scanners, but this gives rise to numerous technical hurdles, physics related concerns, and safety issues in addition to increased exclusiveness. More recently, the development of ultra-low field MR systems has also resurfaced in an effort to focus on accessibility, inherently, avoiding safety issues and wave propagation-related image distortions most typical of high fields. However, their inherently low MR signal amplitude comes at the cost of poor sensitivity.

Both the ultra-high and ultra-low field domains, consequently, call for scientific innovations that pave the way for the next generation of MR hardware systems. Today’s challenges in the development of MR instrumentation arise from the different requirements and limitations faced at different magnetic fields strengths, and all innovations target the improvement of image quality, safety, and/or diagnostic value of MR.

Consequently, this Research Topic has invited submissions on all innovative developments in MR hardware over the entire range of static magnetic field strengths. The resulting collection of articles thus covers a large variety of topics, ranging from ultra-low to ultra-high field MR, including intermediate field strengths, spanning work on radio frequency components, magnet and gradient design, as well as complete MR systems. The Research Topic consists of mostly Original Research articles, augmented by a Perspective and a Mini Review article, as well as a paper in the category Technology and Code.

At ultra-high field strengths, the strongest unmet need concerns the improvement of radiofrequency (RF) transmission systems regarding homogeneity of the RF excitation, efficiency, decoupling strategies, and patient safety with respect to the associated specific absorption rate (SAR) limitations. In this sense, Wenz and Gruetter investigate the impact of quasi-transverse electric modes on the transmit field distribution using dipole-fed rectangular dielectric resonator antennas at 7 T. Also, Williams et al. propose a nested 8-channel transmit array with an open-face concept for human brain imaging at 7 T. In her Mini Review article, Irena Zivkovic discusses available interelement decoupling strategies for ultra-high field MR coils. Van Leeuwen et al. discuss a potential reduction in peripheral local SAR for a birdcage body coil at 3 T using a magnetic shield.

At ultra-low field, but also for MR of X-nuclei (i.e., other than ^1^H), the most challenging task is to increase the sensitivity of the RF detection system, driven by the need to overcome the sensitivity-based limitation of image quality and spatio-temporal resolution set by the various noise sources present in the MR experiment. To this end, several strategies employing new coil geometries, new materials, or cryogenic devices are pursued.

The investigation of novel RF coil element types is an active domain of hardware development for MRI, with the ultimate goal of improving sensitivity. Labbé et al. present their results on high-temperature superconducting RF coils, exploiting the intrinsically low coil noise of such devices. New RF coil shapes are reported by Nowikow et al. in their contribution about Koch Snowflake fractal geometry coils for ^23^Na MRI.

The need for innovative electronics for ultra-low field MR is evidenced by Harper et al. in their work about an unmatched RF chain for low field MRI. In addition, the trend towards portability and ease of use of MR instrumentation calls for other features that increase usability and accessibility but imposes novel technological challenges, on the one hand regarding magnet design for portable MRI systems, and on the other hand flexible, lightweight or wireless RF devices. De Vos et al. introduce an improved portable and sustainable low-field MRI system, complemented by B_0_ shimming methodology for affordable and compact low-field MRI magnets as described by Wenzel et al. In a computational and phantom study at ultra-low field MRI, Höfner et al. investigate the feasibility of 3D neuronal current imaging, relying on the measurement of the minuscule phase perturbation generated by the neuronal magnetic fields.

Regardless of field strength, the development of improved gradient designs enables novel applications of MRI and increases imaging performance. In their Technology and Code article, Littin et al. present an intuitive open-source collection that can be employed to find new gradient coil designs. In an endeavor to reduce acoustic noise and heat in gradient coils, Motovilova and Winkler review existing techniques and present solutions for quiet operation and efficient gradient cooling.

Finally, in a Perspective article, Galuppini et al. discuss the key challenges of field-frequency lock in fast field cycling MRI and define possible research directions in the field.

We, the editors of this Research Topic, hope that the readers will derive added value from the presented collection of articles. We are convinced that innovative hardware concepts and technological development, such as the works presented here, will be highly beneficial for MR research at all field strengths in the future. By combining papers on seemingly very disparate subtopics in MR hardware development into a single collection, we aim at establishing stronger links within the community, so as to foster unconventional and creative solutions for the upcoming challenges in the development and improvement of MR systems.

